# Population-level investigation of the knowledge of ocular chemical injuries and proper immediate action

**DOI:** 10.1186/s13104-020-04950-5

**Published:** 2020-02-25

**Authors:** Hadeel Seraj, Shahad Khawandanh, Arwa Fatani, Afnan Saeed, Ghadeer Alotaibi, Ahmed Basheikh

**Affiliations:** 1 Faculty of Medicine, King Abdulaziz University, Jeddah, Saudi Arabia; 2 Department of Ophthalmology, Faculty of Medicine, King Abdulaziz University, Jeddah, Saudi Arabia; 3 Wasel Aalami SP-630355516, Jeddah, 23622 Saudi Arabia

**Keywords:** Eye damage, Saudi Arabia, Corrective steps, Alkaline, Acidic

## Abstract

**Objective:**

Although the eyes occupy 0.1% of the total body surface, eye injuries are serious because vision is arguably the most important sense. We aimed to assess knowledge of Saudi Arabian residents regarding steps to be taken in cases of chemical eye injury, in order to promote awareness of such procedures.

**Results:**

A cross-sectional design was done. A random sampling method was utilized to select 888 individuals in the Saudi community. Participants completed an electronic closed ended, validated, anonymous, self-administered questionnaire. Two experts assessed content and face validity. Respondents were 18–29 years of age. 74 (8.3%) had a history of chemical eye injury. Regarding the first step taken in case of chemical eye injury, 697 (78.5%) respondents indicated washing with water, 164 (18.5%) indicated visiting the emergency department, 11 (1.2%) indicated using eye drops, and 5 (0.6%) indicated covering the eye immediately. Seventy-five (8.4%) respondents agreed that an eye injured with an acidic material should be washed with an alkaline solution. These results should be confirmed by health authorities and appropriate interventions should be developed for improving knowledge among masses, thereby promoting a healthier society.

## Introduction

The eyes occupy 0.1% of the total body surface and 0.27% of the anterior body surface; however, the seriousness of ocular injury is amplified relative to these proportions, as vision is arguably the most important sense. Loss of vision may affect quality of life because it can lead to job loss, dramatic lifestyle changes, and facial deformities [1, 2]. Ocular injuries place a high burden on the community and are generally preventable [3]. Chemical injuries can involve alkaline and acidic injuries. Alkaline burns are more common, due to the widespread use of alkaline substances in industrial and home cleaning products; these burns typically result in more severe injuries [4]. Approximately 7% of work-related eye injuries treated in hospital emergency departments in the United States are related to chemical injuries [5]. Additionally, more than 60% of chemical injuries occur in workplace accidents, 30% occur at home, and 10% are the result of an accident [6]. Up to 20% of chemical injuries result in massive visual disability and facial disfigurement; notably, visual rehabilitation after an advanced chemical eye injury occurs in less than 15% of the affected individuals [5, 6]. Men are threefold more likely to experience chemical injuries than women; furthermore, individuals aged 16–45 years are most likely to be affected [6].

In Saudi Arabia, a prior study reviewed the data regarding local chemical injuries in two major government hospitals. Among a total of 59 patients (3:1, male:female ratio; mean age, 25 years), alkaline drain cleaners were the most common cause of chemical burns (75% of the patients). In remaining patients, causes comprised concentrated sulfuric acid, car battery acid, and topical application of herbs [7]. Familiarity with various types of ocular and periocular injuries is important for all community members, particularly those living in a predominantly oil—industry driven economy where a large proportion of the population is engaged and handles chemicals frequently. Epidemiological studies are needed to provide information on the rate of knowledge of ocular chemical injuries and proper immediate action. To our knowledge, there have been no investigations about such problem in the general population of Saudi Arabia or elsewhere. So, such study is needed in order to increase awareness of such injuries and to support a healthier society.

## Main text

### Materials and methods

A cross-sectional study was conducted among individuals in the Saudi community during November 2018. A random sampling method was utilized to select 888 individuals. Participants completed an electronic closed ended, validated, anonymous, self-administered open questionnaire. Two experts assessed content and face validity. Internal consistency reliability was 0.81 using alpha Cronbach’s. The questionnaire was distributed online through e-mail and social media websites (Facebook, Twitter, and WhatsApp) Both descriptive and inferential statistics were applied.

The study included both men and women ≥ 18 years of age. Ethical approval was obtained from the Biomedical Ethics Committee at King Abdulaziz University (Jeddah, Saudi Arabia). The sample size was determined by using a sample size calculator site (http://www.raosoft.com/samplesize.html); the minimum recommended sample size was 377, and we included 888 respondents, 74 (8.3%) of which had a history of chemical eye injury. Participants were informed about the length of time of the questionnaire (6–10 min). Furthermore, the purpose of the study was explained and attached to the questionnaire online link. The questionnaire was formulated on the basis of our study objectives. It was tested before fielding via a pilot study and responses were assessed to help refine the questionnaire. Responses were entered manually into a database.

The questionnaire consisted of two parts: the first part acquired demographic data (age, sex, marital status, educational degree, and job); the second part assessed knowledge of ocular chemical injuries and the proper immediate action. Statistical analysis was performed using IBM SPSS Statistics for Windows, Version 21.0. (IBM Corp., Armonk, NY, USA). Descriptive statistics was used. Chi-squared test, odds ratios and 95% confidence intervals were calculated. Frequency and percentages were used to describe categorical variables, while mean (standard deviation) was used to describe continuous numerical variables. P-values of < 0.05 were considered to be statistically significant.

### Results

#### Demographic information

This study included 888 individuals in the Saudi community during November 2018. Most of them were female (624, 70.3%) (see Additional file 1: Table S1).

Age distribution of the respondents is shown with the majority aged 18–29 years (359, 40.4%) (Table 1).

**Table 1:** Age characteristics of the respondents

	Frequency	Percent
18–29	359	40.4
30–39	207	23.3
40–49	150	16.9
50 or more	172	19.4
Total	888	100.0

Regarding education level among the respondents, most (575, 64.8%) had a bachelor’s degree (see Additional file 2: Table S2).

Regarding jobs among the respondents, 234 (26.4%) were students, and 249 (28.0%) worked in the government sector, while 215 (24.2%) were homemakers or unemployed (see Additional file 3: Table S3).

Regarding type of job among the respondents, 286 (32.2%) had an office job, and 160 (18.0%) worked in the medical field (see Additional file 4: Table S4).

A total of 399 (44.9%) respondents selected “not applicable” or “other,” because they were either students or did not have a job, or because of the nature of their work. In our study, 72 (8.1%) respondents had a history of chemical eye injury, including minor food-related injury, such as hot sauce or cooking oil splash. Moreover, 64 (7.2%) respondents had a close friend or family member who experienced a chemical eye injury, including minor food-related injury (Table 2).

**Table 2:** Question: Do you or your acquaintances have a history of chemical eye injury?

	Frequency	Percent
Yes (self)	74	8.3
Yes (close friend or family member)	64	7.2
No	750	84.5
Total	888	100

#### Knowledge related to chemical eye injuries

In total, 784 (88.3%) respondents agreed that chemical substances can cause eye complications (Table 3). Regarding the first corrective action in case of chemical eye injury, majority of the respondents (697, 78.5%) said that washing the eye with water should be the first action; of these 697 respondents, 655 (73.8%) said that washing the eye with *plenty* of water should be performed, and 42 (4.7%) said that washing the eye with *little* water was appropriate. In contrast, 164 (18.5%) respondents said that going to the emergency department should be the first corrective action (Table 3).

**Table 3:** Answers to questions and statements testing the knowledge related to chemical eye injuries

	Frequency	Percent
Statement: chemical injury can cause ocular complications		
Agree	784	88.3
Disagree	14	1.6
Don’t know	90	10.1
Total	888	100.0
Question: what should be the first corrective action when a chemical injury occurs?		
Wash with plenty of water	655	73.8
Wash with little of water	42	4.7
Cover the eye	5	0.6
Go to emergency department	164	18.5
Pharmacy and eye drops	11	1.2
Other	11	1.2
Total	888	100.0
Statement: alkaline injuries are more dangerous than acidic injuries		
Agree	491	55.3
Disagree	140	15.8
Don’t know	257	28.9
Total	888	100.0
Statement: locate and remove particles in the eye in case of chemical injury		
Agree	552	62.2
Disagree	153	17.2
Don’t know	183	20.6
Total	888	100.0
Statement: when injured with acidic material, wash with alkaline solution		
Agree	75	8.4
Disagree	353	39.8
Don’t know	460	51.8
Total	888	100.0
Statement: when injured with alkaline material, wash with acid solution		
Agree	60	6.8
Disagree	404	45.5
Don’t know	424	47.7
Total	888	100.0

Regarding whether alkaline injuries are more dangerous than acidic injuries, 491 (55.3%) respondents agreed, 140 (15.8%) disagreed, and 257 (28.9%) did not know (Table 3). Regarding whether particles should be located and removed in case of chemical injury, 552 (62.2%) respondents agreed (Table 3). Regarding whether an eye injured with an acidic substance should be washed with an alkaline solution, 75 (8.4%) respondents agreed, 353 (39.8%) disagreed, and 460 (51.8%) did not know (Table 3). Regarding whether an eye injured with an alkaline substance should be washed with an acidic solution, 60 (6.8%) respondents agreed, 404 (45.5%) disagreed, and 424 (47.7%) did not know (Table 3).

### Discussion

Vision is one of the most important human functions. Loss of vision from chemical injury may greatly affect quality of life. This study aimed to assess the knowledge of immediate corrective action in cases of chemical eye injury, among individuals in the Saudi community, in order to reduce the incidence of ocular injuries and related complications. When our participants were asked whether chemical substances can cause eye complications, we found that 88.3% agreed; this is consistent with the findings of another study conducted among Latino farm workers, where 97.3% agreed that wind, dust, and chemicals could cause eye problems [8].

In cases of chemical ocular injuries, rapid irrigation and dilution of the chemical with water should be the immediate first corrective action, in order to reduce tissue damage and protect vision [9, 10]. In our study, 78.5% of the respondents indicated that washing the eyes with water should be the first corrective action. However, 8.4% of respondents in our study indicated that in cases of acidic eye injuries, the eyes should be washed with an alkaline solution, which is extremely dangerous; moreover, 6.8% indicated the opposite washing regimen, that eyes should be washed with an acidic solution in cases of alkaline eye injuries, which is also dangerous. Approximately 50% of our respondents did not know that both the washing regimens could be dangerous and therefore, should not be considered at all.

The severity of ocular injury is related to the type of chemical, the volume and pH (alkaline or acidic) of the solution, and the duration of exposure. Notably, alkaline solutions penetrate more rapidly into the eye, relative to acids; therefore, alkaline solutions are more damaging to intraocular structures such as the iris, lens, and ciliary body, resulting in rapid and irreversible damage [11]. In our study, 55.3% of the respondents agreed that alkaline eye injuries were more dangerous than acidic eye injuries. After ocular chemical injury, it is important to search for and remove solid particles that could be trapped in the conjunctival fornices and act as reservoir for continued chemical release and inflammation [6, 9]. This was recognized by 62.2% of the respondents in our study, who agreed that particles should be located and removed in case of chemical eye injury.

### Conclusion

The eye is an important, irreplaceable organ. Loss of vision through ocular chemical injury is a serious condition that can negatively affect quality of life, primarily through job loss and increased dependence on others. Our findings indicate that individuals in the Saudi population need greater awareness regarding immediate corrective action in cases of ocular chemical injuries. Furthermore, there is a need to clearly communicate that water should be the only solution used to irrigate the eye; no other solutions should be used, regardless of pH. Expansion of these results should be performed by health authorities, in conjunction with the development of appropriate interventions, such as health awareness campaigns regarding ocular chemical injuries and immediate corrective actions, in order to improve knowledge and to create a healthier society.

## Limitation

The primary limitation to the generalization of these results is the selection bias. We used an electronic questionnaire for our study; therefore, the results are subject to the bias that people with higher level of education (that could answer the questionnaire) were included. Future studies are needed that should include a wider section of population irrespective of the level of education.

## Supplementary information

**Additional file 1: Table S1.** Sex characteristics of the respondents. Most of our study participants were female (624, 70.3%).

**Additional file 2: Table S2.** Educational level of the respondents. Most of the respondents had a bachelor’s degree (575, 64.8%).

**Additional file 3: Table S3.** Jobs of the respondents. About 234 (26.4%) respondents were students, and 249 (28.0%) worked in the government sector, while 215 (24.2%) were homemakers or unemployed.

**Additional file 4: Table S4.** Type of jobs among respondents. About 286 (32.2%) respondents had an office job, and 160 (18.0%) worked in the medical field.

## Data Availability

The data are not available for public access because of privacy concerns; however, these could be obtained from the corresponding author on reasonable request.
