# Supplementary material for: Population-level investigation of the knowledge of ocular chemical injuries and proper immediate action
Source: BMC Res Notes. 2020 Feb 25;13:103. doi: 10.1186/s13104-020-04950-5 (PMC7043023; doi:10.1186/s13104-020-04950-5)
Supplement: Supplementary file 1 — Additional file 1: Table S1. Sex characteristics of the respondents. Most of our study participants were female (624, 70.3%). [file 13104_2020_4950_MOESM1_ESM.docx]

**Additional table 1. Sex characteristics of the respondents**

|  | **Frequency** | **Percent** |
| --- | --- | --- |
| **Male** | 264 | 29.7 |
| **Female** | 624 | 70.3 |
| **Total** | 888 | 100.0 |
