# Supplementary material for: Population-level investigation of the knowledge of ocular chemical injuries and proper immediate action
Source: BMC Res Notes. 2020 Feb 25;13:103. doi: 10.1186/s13104-020-04950-5 (PMC7043023; doi:10.1186/s13104-020-04950-5)
Supplement: Supplementary file 2 — Additional file 2: Table S2. Educational level of the respondents. Most of the respondents had a bachelor’s degree (575, 64.8%). [file 13104_2020_4950_MOESM2_ESM.docx]

**Additional table 2. Educational level of the respondents**

|  | **Frequency** | **Percent** |
| --- | --- | --- |
| **Elementary** | 15 | 1.7 |
| **Intermediate** | 12 | 1.4 |
| **High School** | 173 | 19.5 |
| **Bachelor’s** | 575 | 64.8 |
| **Master’s** | 73 | 8.2 |
| **PhD** | 36 | 4.1 |
| **Other** | 4 | 0.5 |
| **Total** | 888 | 100.0 |
