# Supplementary material for: Population-level investigation of the knowledge of ocular chemical injuries and proper immediate action
Source: BMC Res Notes. 2020 Feb 25;13:103. doi: 10.1186/s13104-020-04950-5 (PMC7043023; doi:10.1186/s13104-020-04950-5)
Supplement: Supplementary file 3 — Additional file 3: Table S3. Jobs of the respondents. About 234 (26.4%) respondents were students, and 249 (28.0%) worked in the government sector, while 215 (24.2%) were homemakers or unemployed. [file 13104_2020_4950_MOESM3_ESM.docx]

**Additional table 3. Jobs** **of the respondents**

|  | **Frequency** | **Percent** |
| --- | --- | --- |
| **Student** | 234 | 26.4 |
| **Government** | 249 | 28.0 |
| **Private** | 136 | 15.3 |
| **Not working/homemaker** | 215 | 24.2 |
| **Self-employed** | 20 | 2.3 |
| **Other** | 34 | 3.8 |
| **Total** | 888 | 100.0 |
