# Supplementary material for: Population-level investigation of the knowledge of ocular chemical injuries and proper immediate action
Source: BMC Res Notes. 2020 Feb 25;13:103. doi: 10.1186/s13104-020-04950-5 (PMC7043023; doi:10.1186/s13104-020-04950-5)
Supplement: Supplementary file 4 — Additional file 4: Table S4. Type of jobs among respondents. About 286 (32.2%) respondents had an office job, and 160 (18.0%) worked in the medical field. [file 13104_2020_4950_MOESM4_ESM.docx]

**Additional table 4. Type of jobs among respondents**

|  | **Frequency** | **Percent** |
| --- | --- | --- |
| **Office** | 286 | 32.2 |
| **Manufacturing** | 16 | 1.8 |
| **Construction/building** | 12 | 1.4 |
| **Agriculture** | 3 | 0.3 |
| **Medical** | 160 | 18.0 |
| **Soldier** | 12 | 1.4 |
| **N/A** | 375 | 42.2 |
| **Other** | 24 | 2.7 |
| **Total** | 888 | 100.0 |
